# New-onset postoperative atrial fibrillation after pulmonary endarterectomy is associated with adverse outcomes

**DOI:** 10.3389/fsurg.2024.1380570

**Published:** 2024-05-30

**Authors:** Dingkai Zhang, Zhaohua Zhang, Yanan Zhen, Xiaopeng Liu, Xueqiang Fan, Zhidong Ye, Peng Liu

**Affiliations:** ^1^ China-Japan Friendship Hospital (Institute of Clinical Medical Sciences), Chinese Academy of Medical Sciences & Peking Union Medical College, Beijing, China; ^2^ Department of Cardiovascular Surgery, China-Japan Friendship Hospital, Beijing, China; ^3^ Department of Cardiovascular Surgery, Peking University China-Japan Friendship School of Clinical Medicine, Beijing, China

**Keywords:** postoperative atrial fibrillation (POAF), risk factors, adverse outcomes, chronic thromboembolic pulmonary hypertension (CTEPH), pulmonary thromboendarterectomy (PEA)

## Abstract

**Background:**

New-onset postoperative atrial fibrillation (POAF) is a common complication after pulmonary thromboendarterectomy (PEA), yet the risk factors and their impact on prognosis remain poorly understood. This study aims to investigate the risk factors associated with new-onset POAF after PEA and elucidate its underlying connection with adverse postoperative outcomes.

**Methods:**

A retrospective analysis included 129 consecutive chronic thromboembolic pulmonary hypertension (CTEPH) patients and 16 sarcoma patients undergoing PEA. Univariate and multivariate analyses were conducted to examine the potential effects of preoperative and intraoperative variables on new-onset POAF following PEA. Propensity score matching (PSM) was then employed to adjust for confounding factors.

**Results:**

Binary logistic regression revealed that age (odds ratio [OR] = 1.041, 95% confidence interval [CI] = 1.008–1.075, *p = *0.014) and left atrial diameter[LAD] (OR = 1.105, 95% CI = 1.025–1.191, *p* = 0.009) were independent risk factors for new-onset POAF after PEA. The receiver operating characteristic (ROC) curve indicated that the predictive abilities of age and LAD for new-onset POAF were 0.652 and 0.684, respectively. Patients with new-onset POAF, compared with those without, exhibited a higher incidence of adverse outcomes (in-hospital mortality, acute heart failure, acute kidney insufficiency, reperfusion pulmonary edema). Propensity score matching (PSM) analyses confirmed the results.

**Conclusion:**

Advanced age and LAD independently contribute to the risk of new-onset POAF after PEA. Patients with new-onset POAF are more prone to adverse outcomes. Therefore, heightened vigilance and careful monitoring of POAF after PEA are warranted.

## 1 Introduction

Chronic thrombotic pulmonary hypertension (CTEPH) originates from organized thrombosis and fibrous stenosis in the pulmonary artery, leading to reduced exercise capacity, dyspnea, and progressive right heart failure, and is Categorized as Group 4 pulmonary hypertension by the World Health Organization (WHO) (1), CTEPH poses severe life-threatening consequences, with a low survival rate if untreated. Patients with mean pulmonary artery pressure (mPAP) >40 mmHg exhibit a 5-year survival of 30%, and the 5-year survival is only 10% among those with mPAP values exceeding 50 mmHg (2). Approximately 0.1 to 9% of survivors of pulmonary embolism eventually develop CTEPH (3), and nearly a quarter of CTEPH patients have no definite history of prior pulmonary embolism (4). The atypical early symptoms of CTEPH often lead to overlooked or misdiagnosed cases, suggesting that the incidence of CTEPH may be higher than reported in existing research (5).

Pulmonary thromboendarterectomy (PEA) stands as a potentially curative treatment for CTEPH by removing obstructions from the main pulmonary artery up to subsegmental levels, significantly improving patient survival compared to medical therapies (6). However, PEA is complex and challenging, associated with a high risk of mortality and complications (3, 7–9). Postoperative atrial fibrillation (POAF), a common occurrence in cardiac surgery, profoundly affects the postoperative recovery and prognosis (10). Some previous studies have confirmed that a percentage of patients experience atrial arrhythmias following PEA (11, 12). Despite this, existing studies have not comprehensively elucidated the risk factors related to new-onset atrial fibrillation after PEA, and there is a notable gap in research on the impact of new-onset POAF on early prognosis. This study includes common complications after PEA as adverse outcomes (in-hospital mortality, acute heart failure, acute kidney insufficiency, and reperfusion pulmonary edema). The primary objective is to assess relevant risk factors associated with new-onset POAF in patients undergoing PEA and to evaluate the influence of new-onset POAF on early postoperative outcomes.

## 2 Methods

### 2.1 Study population and design

**Figure 1:**
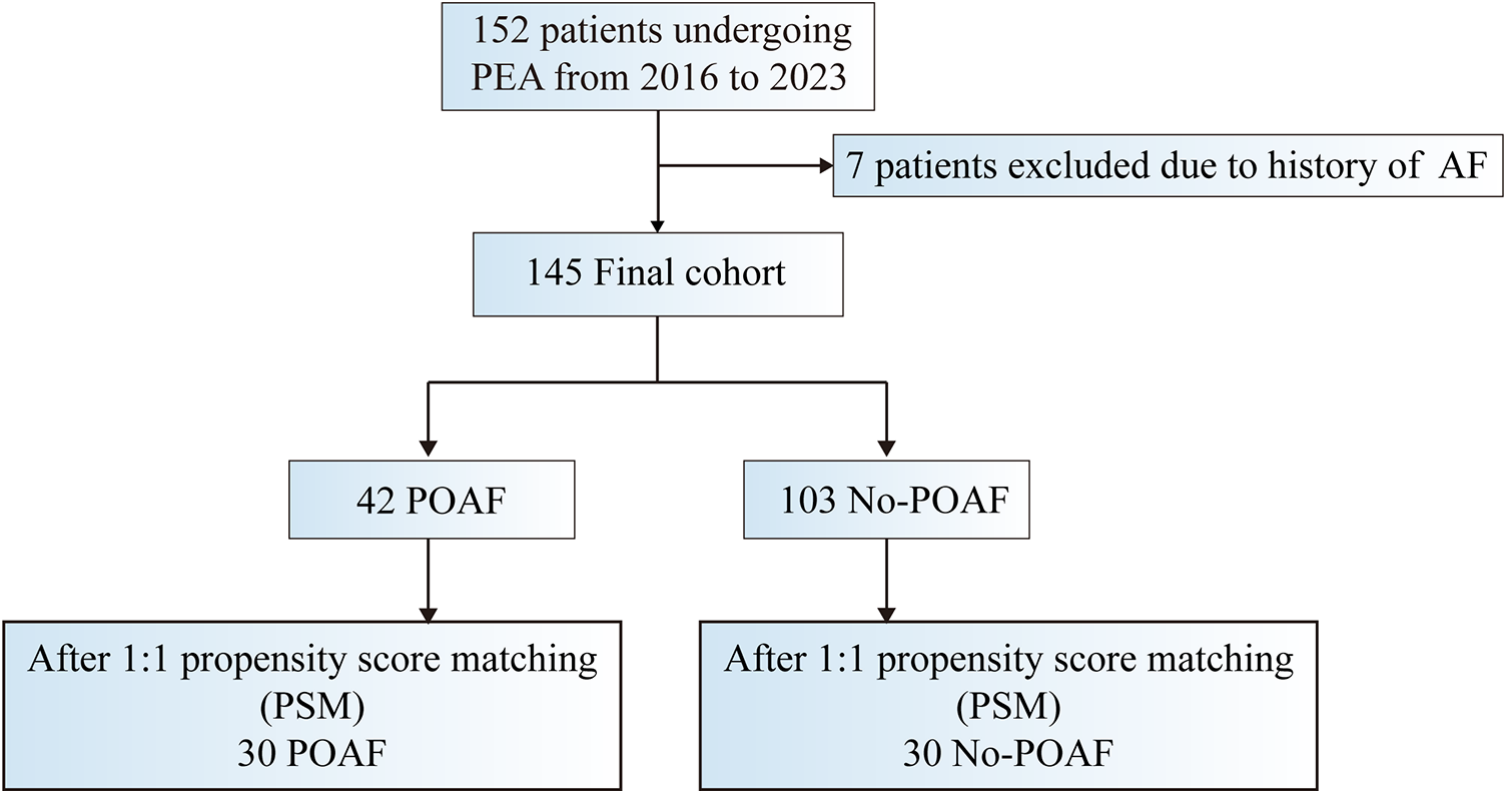
Study design: summary of inclusion and exclusion criteria. AF indicates atrial fibrillation; POAF postoperative atrial fibrillation; PSM, propensity score matching; and PEA, pulmonary endarterectomy.

This retrospective, observational cohort study enrolled patients who underwent pulmonary endarterectomy at our hospital between December 2016 and April 2023. Illustrated in Figure 1, a total of 152 patients undergoing PEA were initially included. After excluding 7 patients with a history of paroxysmal or persistent atrial fibrillation or atrial flutter before surgery, the research focused on 145 patients, each confirmed to have sinus rhythm through routine electrocardiogram (ECG) before surgery. Among these, 129 were diagnosed with CTEPH, and 16 were diagnosed with sarcoma. The study adheres to the Declaration of Helsinki and received approval from the ethics board of the China-Japan Friendship Hospital, waiving the requirement for informed consent as the data were obtained from routine patient care, used for clinical purposes, and handled anonymously.

### 2.2 Data sources

All surgeries were conducted by a consistent team of surgeons, utilizing cardiopulmonary bypass with deep hypothermia circulatory arrest. Following the procedure, patients were transitioned to the surgical care unit for subsequent management. Postoperative ECG was obtained from both ECG monitoring and telemetry ECG monitoring. Continuous monitoring of the cardiac rhythm was employed in the ICU ward, utilizing a bedside ECG monitor or telemetry ECG monitor for recording the patient's cardiac rhythm upon transfer to the general ward. Patient data were systematically collected through the electronic medical record system.

### 2.3 Clinical variables definition

#### 2.3.1 Definition of new-onset POAF

Following the definition provided by the Society of Thoracic Surgeons National database (13), new-onset POAF was characterized by the presence of atrial fibrillation or atrial flutter persisting for a minimum of 5 min after PEA. Patients maintaining a consistent sinus rhythm were categorized into the sinus rhythm (Non-POAF) group, which was assessed five days post-surgery. Amiodarone was administered to patients experiencing a single episode of atrial fibrillation lasting over 20 min or accumulating a 24-hour duration exceeding 1 h.

#### 2.3.2 Definition of acute heart failure

The diagnosis of acute heart failure was conducted by a cardiologist who integrated clinical symptoms, signs, and measurements.

#### 2.3.3 Definition of acute kidney insufficiency

Acute kidney insufficiency was characterized by a serum creatinine increase exceeding 1.5 times baseline values, a glomerular filtration rate decrease exceeding 25%, or urine output less than 0.5 ml/kg/h for a duration of 6 h.

#### 2.3.4 Definition of reperfusion pulmonary edema

The diagnostic criteria for RPE included exudation in chest x-ray, a chest radiograph score greater than 1, a PaO_2_/FiO_2_ ratio less than 300 mmHg, and the exclusion of other potential causes such as atelectasis or pneumonia.

#### 2.3.5 Definition of stoke

A persistent central neurologic deficit (focal or generalized) was diagnosed as stroke, as assessed by 1 neurologist who integrated clinical symptoms, signs, and imaging materials.

### 2.4 Anticoagulants

Oral anticoagulants are routine treatment for all patients with CTEPH. According to the different mechanisms of the drugs, they were divided into two categories: vitamin K antagonists(VKAs) and direct oral anticoagulants (DOACs).

### 2.5 Statistical analysis

Continuous variables displaying a normal distribution were expressed as mean ± standard deviation, while non-normally distributed continuous variables were presented as median (inter-quartile range). Categorical variables were conveyed as the number of cases or percentages. An unpaired *t*-test or the Mann-Whitney test was employed for continuous variables, and the Chi-square test or Fisher exact test was utilized for comparing categorical variables. Variables with a *p*-value <0.05 from univariate analysis were chosen for inclusion in binary logistic regression analysis. The Receiver Operating Characteristic curve (ROC) and the Youden index were employed to ascertain optimal cutoff values for continuous variables. The Area Under the Curve (AUC) was used to assess the diagnostic capability of risk factors. Statistical analyses were conducted using SPSS (version 22.0, IBM Corp.) and R (4.3.1). All tests were two-tailed, and a significance threshold of *p *< 0.05 was considered.

## 3 Results

### 3.1 Patient characteristics

A total of 42 (29.0%) patients were diagnosed with new-onset POAF in this cohort population. The baseline characteristics of the patients are detailed in Table 1. In comparison with the Non-POAF group, patients in the POAF group were older, exhibited a higher prevalence of hypertension, had shorter distances in the 6-minute walking test (6MWT), demonstrated worse WHO functional class grades, and presented with a larger left atrial diameter (LAD) (Table 1). Intraoperative characteristics of the patients are outlined in Table 2. There were no significant differences observed between patients with and without POAF in intraoperative characteristics (Table 2).

**Table 1:** Preoperative characteristics in patients with and without new-onset POAF.

Characteristics	Non-POAF group (*n* = 103)	POAF group (*n* = 42)	Statistics	*P*
Male (*n*, %)	63 (61.17)	28 (66.67)	0.386	0.534
Age (years)	47.87 ± 14.19	55.10 ± 10.66	−2.972	0.003
BMI (kg/m^2^)	23.93 ± 3.41	24.60 ± 3.33	−1.080	0.282
Smoking (*n*, %)	38 (36.89)	16 (38.10)	0.018	0.892
DOACs (*n*, %)	46 (44.66)	23 (54.76)	1.221	0.269
DVT (*n*, %)	28 (27.18)	13 (30.95)	0.209	0.648
Diabetes (*n*, %)	3 (2.91)	4 (9.52)	1.582	0.209
Dyslipidemia (*n*, %)	39 (37.86)	10 (23.81)	2.634	0.105
Hypertension (*n*, %)	18 (17.48)	15 (35.71)	5.646	0.018
6MWT (m)	376.01 ± 136.01	300.52 ± 170.87	2.555	0.013
BNP (pg/ml)	1,419.87 ± 3,623.48	1,524.14 ± 1,873.04	−0.177	0.860
Heart rate	76.44 ± 7.17	76.05 ± 8.63	0.279	0.781
PVR (dynes·s·cm^−5^)	865.74 ± 506.52	848.19 ± 536.14	0.186	0.853
mPAP (mmHg)	39.61 ± 14.09	39.24 ± 12.91	0.148	0.882
TAPSE (mm)	17.38 ± 4.01	16.92 ± 3.93	0.633	0.528
TAPSV (cm/s)	10.47 ± 2.68	10.23 ± 2.41	0.505	0.615
COPD (*n*, %)	17 (16.50)	12 (28.57)	2.715	0.099
WHO functional class (*n*, %)			−2.654	0.008
Ⅱ	52 (50.49)	13 (30.95)		
Ⅲ	41 (39.81)	18 (42.86)		
Ⅳ	10 (9.71)	11 (26.19)		
EF	60.47 ± 5.19	59.55 ± 5.33	0.959	0.339
LAD (mm)	33.94 ± 5.28	36.95 ± 4.71	−3.209	0.002
LVEDD (mm)	42.61 ± 5.41	43.55 ± 5.54	−0.939	0.349
LVESD (mm)	25.76 ± 3.98	26.00 ± 3.96	−0.333	0.739

BMI, body mass index; DOACs, direct oral anticoagulants; DVT, deep vein thrombosis; BNP, brain natriuretic peptide; 6MWT, 6-minute walking test; PVR, pulmonary vascular resistance; mPAP, mean pulmonary artery pressure; TAPSE, tricuspid annular plane systolic excusion; TAPSV, tricuspid annular peak systolic velocity; COPD, chronic obstructive pulmonary disease; WHO, World Health Organization; EF, ejection fraction; LAD, left atrial diameter; LVEDD, left ventricular end-diastolic dimension; LVESD, left ventricular end-systolic dimension.

**Table 2:** Intraoperative characteristics in patients with and without new-onset POAF.

Characteristics	Control group (*n* = 103)	Observation group (*n* = 42)	Statistics	*P*
Operation time (min)	583.91 ± 86.57	594.41 ± 85.61	−0.664	0.508
Duration of CPB (min)	345.22 ± 63.76	350.64 ± 81.89	−0.426	0.671
Duration of aorta clamping (min)	161.11 ± 39.38	160.45 ± 40.56	0.090	0.928
Circulatory arrest time (min)	56.78 ± 20.08	55.51 ± 20.76	0.342	0.733
Times of Circulatory arrest	3.42 ± 1.11	3.17 ± 1.17	1.265	0.208

CPB, cardiopulmonary bypass.

### 3.2 Predictors of new-onset POAF

Variables with *p *< 0.05 (age, hypertension, 6MWT, WHO functional class, LAD) were incorporated into the binary logistic regression model. Age (odds ratio [OR] = 1.041, 95% confidence interval [CI] = 1.008–1.075, *p = *0.014) and LAD (odds ratio [OR] = 1.105, 95% confidence interval [CI] = 1.025–1.191, *p* = 0.009) were identified as independent risk factors for new-onset POAF (Table 3).

**Table 3:** Multivariate risk factors for new-onset POAF.

Risk factor	OR	95% CI	*P*
Age (years)	1.041	1.008–1.075	0.014
LAD (mm)	1.105	1.025–1.191	0.009

**Figure 2:**
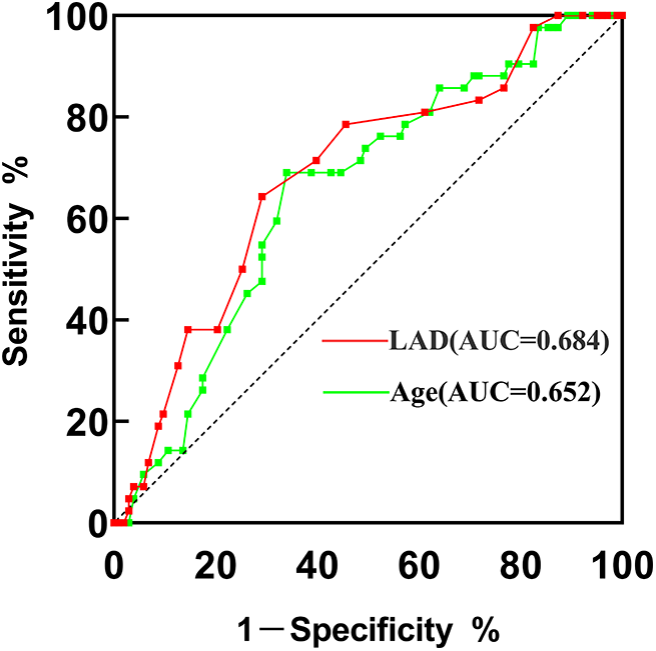
ROC curve of VWF and age for predicting new-onset POAF after PEA. AUC, area under the curve; PEA, pulmonary endarterectomy; ROC, receiver operating characteristic.

The ROC curves illustrated that the AUC of age and LAD were 0.652 (95% CI = 0.558–0.747, *p *= 0.004) and 0.684 (95% CI = 0.590–0.779, *p *= 0.001), respectively (Figure 2). The optimal cutoff point of age was 54.5, with a sensitivity of 69.0% and a specificity of 66.0%. For LAD, the cutoff value was 35.5, with a sensitivity of 64.3%, and specificity of 70.9%.

### 3.3 Clinical outcomes

Patients with new-onset POAF exhibited prolonged length of stay, mechanical ventilation, and intensive care unit occupancy. Moreover, they demonstrated elevated in-hospital mortality (23.81% vs. 1.94%, *p *< 0.001), along with an increased occurrence of complications such as acute kidney injury, reperfusion pulmonary edema, and acute heart failure. The rate for stroke was higher in the POAF group than another group(without POAF), but the difference was not statistically significant (Table 4).

**Table 4:** Postoperative characteristics in patients with and without new-onset POAF.

Characteristics	Control group (*n* = 103)	Observation group (*n* = 42)	Statistics	*P*
Hospital length of stay, d	19.84 ± 8.11	25.14 ± 14.07	−2.294	0.026
Intensive care unit time, d	5.57 ± 4.63	12.55 ± 13.32	−3.312	0.002
Mechanical ventilation time, h	60.86 ± 50.09	151.16 ± 145.53	−3.928	<0.001
In-hospital mortality, *n* (%)	2 (1.94)	10 (23.81)	16.024	<0.001
Acute kidney injury, *n* (%)	24 (23.30)	22 (52.38)	11.648	0.001
Reperfusion Pulmonary Edema, *n* (%)	15 (14.56)	15 (35.71)	8.134	0.004
Acute heart Failure, *n* (%)	18 (17.48)	23 (54.76)	20.452	<0.001
Stroke, *n* (%)	4 (3.88)	3 (7.14)	0.163	0.687
Re-exploration, *n* (%)	2 (1.94)	2 (4.76)	–	0.580

### 3.4 Propensity score matching (PSM)

To account for baseline disparities and validate the findings, we conducted propensity score matching (PSM) for 30 patients with POAF and 30 without (Table 5). Even after PSM, the POAF group continued to exhibit an extended duration of mechanical ventilation, elevated in-hospital mortality (26.67% vs. 3.33%, *p = *0.030), and a heightened frequency of complications, including acute kidney injury, reperfusion pulmonary edema, and acute heart failure (Table 6).

**Table 5:** Baseline characteristics following propensity score matching (PSM).

Characteristics	Non-POAF group (*n* = 30)	POAF group (*n* = 30)	*P*
Male (*n*, %)	17 (56.67)	20 (66.67)	0.595
Age (years)	57.27 ± 10.00	54.13 ± 11.19	0.258
BMI (kg/m^2^)	24.30 ± 3.73	24.65 ± 3.61	0.717
Smoking (*n*, %)	14 (46.67)	12 (40.00)	0.794
DVT (*n*, %)	6 (20.00)	8 (26.67)	0.760
DOACs (*n*,%)	13 (43.33)	16 (53.33)	0.438
Diabetes (*n*, %)	2 (6.67)	4 (13.33)	0.667
Dyslipidemia (*n*, %)	10 (33.33)	8 (26.67)	0.778
Hypertension (*n*, %)	9 (30.00)	9 (30.00)	1.000
6MWT (m)	342.43 ± 125.32	324.90 ± 173.97	0.656
BNP (pg/ml)	2,213.92 ± 6,349.33	1,506.60 ± 2,093.78	0.565
Heart rate	75.63 ± 8.43	75.10 ± 8.01	0.803
PVR (dynes·s·cm^−5^)	887.99 ± 542.53	855.29 ± 589.18	0.824
mPAP (mmHg)	38.77 ± 11.51	39.47 ± 13.08	0.827
TAPSE (mm)	16.73 ± 4.22	17.18 ± 3.68	0.664
TAPSV (cm/s)	10.27 ± 2.55	10.41 ± 2.47	0.834
COPD (*n*, %)	5 (16.67)	8 (26.67)	0.531
WHO functional class (*n*, %)			0.478
Ⅱ	11 (36.67)	12 (40.00)	
Ⅲ	15 (50.00)	11 (36.67)	
Ⅳ	4 (13.33)	7 (23.33)	
EF (%)	60.90 ± 5.60	59.13 ± 5.13	0.208
LAD (mm)	35.37 ± 6.31	36.13 ± 4.81	0.137
LVEDD (mm)	41.47 ± 6.16	43.37 ± 5.83	0.225
LVESD (mm)	25.27 ± 4.27	25.80 ± 4.33	0.633
Operation time (min)	589.30 ± 75.44	593.67 ± 97.15	0.847
Duration of CPB (min)	337.57 ± 58.87	348.53 ± 93.70	0.589
Duration of aorta clamping (min)	157.73 ± 33.48	152.43 ± 36.90	0.562
Circulatory arrest time (min)	57.58 ± 21.11	53.55 ± 16.94	0.418
Times of Circulatory arrest	3.50 ± 1.22	3.07 ± 0.98	0.136

BMI, body mass index; DOACs, direct oral anticoagulants; DVT, deep vein thrombosis; BNP, brain natriuretic peptide; 6MWT, 6-minute walking test; PVR, pulmonary vascular resistance; mPAP, mean pulmonary artery pressure; TAPSE, tricuspid annular plane systolic excusion; TAPSV, tricuspid annular peak systolic velocity; COPD, chronic obstructive pulmonary disease; WHO, World Health Organization; EF, ejection fraction; LAD, left atrial diameter; LVEDD, left ventricular end-diastolic dimension; LVESD, left ventricular end-systolic dimension.

**Table 6:** Postoperative outcomes following propensity score matching (PSM).

Characteristics	Control group (*n* = 30)	Observation group (*n* = 30)	Statistics	*P*
Hospital length of stay, d	21.07 ± 7.33	25.00 ± 15.17	−1.279	0.208
Intensive care unit time, d	7.63 ± 5.92	12.20 ± 14.23	−1.623	0.113
Mechanical ventilation time, h	83.88 ± 53.26	144.31 ± 153.18	−2.041	0.049
In-hospital mortality, *n* (%)	1 (3.33)	8 (26.67)	4.706	0.030
Acute kidney injury, *n* (%)	6 (20.00)	15 (50.00)	5.934	0.015
Reperfusion Pulmonary Edema, *n* (%)	2 (6.67)	9 (30.00)	4.007	0.045
Acute heart Failure, *n* (%)	4 (13.33)	14 (46.67)	6.429	0.011
Stroke, *n* (%)	2 (6.67)	3 (10.00)	-	1.000

## 4 Discussion

As the most prevalent arrhythmia following cardiac surgery, new-onset POAF has an incidence ranging from 20% to 50%, often closely linked to adverse outcomes (14, 15). However, evidence for POAF after PEA is insufficient, and previous research lacks definitive standards for POAF. In the Farasat et al. study, POAF was defined as the presence of any atrial arrhythmias based on telemetry findings (11). Conversely, another report recorded various electrocardiography results for patients at 1–6 monthly intervals after PEA, excluding those who developed atrial fibrillation/atrial tachycardia only in the first 30 days post-surgery (12). Our study differs from previous ones as it excluded patients with preoperative atrial arrhythmia, focusing specifically on the new-onset POAF after PEA. Given that POAF typically manifests between days 2 and 4 postoperatively (16), we recorded atrial fibrillation or atrial flutter occurrences five days after surgery as the criterion. In this study, the incidence of new-onset POAF after PEA was 29.0%, aligning closely with the results of previous studies (11, 12, 17).

Advanced age has been consistently identified as an independent risk factor for POAF in numerous clinical studies (18–21). Despite our patient group not surpassing the age of those reported in the cardiac surgery literature, we observed that older age remains a significant risk factor for atrial fibrillation after PEA. The aging process contributes not only to the loss of myocardial fibers but also exacerbates fibrosis and collagen deposition in the atria, particularly near the sinoatrial node, impacting atrial electrical properties (15). These age-related pathophysiological changes constitute fundamental factors in triggering POAF.

Left atrial enlargement is established as another risk factor for the occurrence of new-onset POAF after cardiovascular surgery, as corroborated by numerous prior studies (6, 7). Although pathologic changes in CTEPH primarily affect the right cardiac system, our findings reaffirm that an enlarged LAD is once again identified as a risk factor for POAF. The enlarged atrium induces remodeling of its electrophysiological conduction pathways, resulting in increased irritability of cardiomyocytes, shortened atrial refractory periods, delayed conduction, enhanced extracellular matrix fibrosis, heightened heterogeneity of atrial repolarization, and ultimately promoting the onset of atrial fibrillation.

PEA stands as the exclusive curative option for CTEPH and is recommended for patients suitable for surgical intervention. Nevertheless, it remains a challenging, invasive, and time-intensive procedure, often accompanied by common postoperative complications such as acute heart failure, acute kidney insufficiency, and reperfusion pulmonary edema (22, 23). Consequently, we designated the aforementioned three complications and in-hospital mortality as adverse outcomes. Across both cardiac and non-cardiac surgery, nearly all studies have consistently underscored the association between POAF and adverse outcomes (15–26). POAF is intricately linked to thrombosis, heart failure, stroke, and increased mortality. Our findings also reveal that patients with POAF experience a more adverse prognosis during the early postoperative period. While there was no statistically significant difference in the length of hospital and ICU stays between the two groups after PSM, patients with POAF remained predisposed to developing acute heart failure, acute kidney insufficiency, reperfusion pulmonary edema, and in-hospital mortality compared to the control group. Atrial fibrillation disrupts the hemodynamics, not only escalating the burden on the heart but also severely impairing the pumping function, leading to inadequate perfusion of vital organs such as the kidneys and lungs. Hence, it is unsurprising that corresponding damage ensues. Although our study did not observe the impact of POAF on long-term outcomes in these patients, some research has indicated that POAF also significantly influences postoperative long-term prognosis (27–29). Thus, it is imperative to identify the risk factors of POAF and implement preventive measures accordingly.

Furthermore, patients in the POAF group experienced prolonged mechanical ventilation, even after PSM. Previous studies have indicated that extended ventilation serves as a predictor of POAF (30). This is primarily due to the fact that, on the one hand, patients requiring prolonged mechanical ventilation typically exhibit more severe conditions, compromised cardiopulmonary function, and an increased likelihood of postoperative atrial fibrillation. On the other hand, mechanical ventilation induces alterations in pleural and thoracic pressure, as well as lung capacity, influencing changes in preload, afterload, heart rate, and myocardial contractility. Variations in intrathoracic pressure directly impact the heart, pericardial cavity, major arteries, and veins. Spontaneous breathing generates intrathoracic negative pressure, lowering the pressure in the right atrium. Conversely, intermittent positive pressure ventilation (IPPV) elevates intrathoracic pressure and right atrium pressure, while positive end-expiratory pressure (PEEP) maintains intrathoracic pressure consistently higher than atmospheric pressure throughout the respiratory cycle. Thus, these factors affecting right atrial pressure may contribute to the onset of POAF. However, acknowledging the bidirectional relationship between mechanical ventilation and atrial fibrillation, we treated mechanical ventilation as a postoperative observation rather than an analytical factor. Nonetheless, PEA is a complex and demanding procedure, often involving extended CPB durations and deep hypothermic circulatory arrest. These factors may induce heightened stress and inflammation, influencing the mechanisms underlying POAF. While previous studies have consistently associated CPB and aortic cross-clamp durations with POAF in cardiac surgery (31, 32), we did not observe a similar outcome in our study.

Some other factors may also trigger the occurrence of POAF. A recent research revealed that Del Nido cardioplegia can significantly reduced POAF rates after coronary artery bypass grafting, compared with cold blood cardioplegia. The occurrence of POAF is associated with electrolyte disorders or “retained blood syndrome”. Some reports indicated that the inotropic drugs could increase the risk of POAF (33, 34). Del Nido cardioplegia have an advantage on the electrolyte balance, myocardial protection and the need for postoperative inotropic support. It's a positive role on the volemic balance result in less occurrence of POAF (35). However, as a single center, our team's practice for PEA, which requires prolonged aortic occlusion, is always to use HTK solution as cardioplegia. Therefore, we can't observe the effect of this factor. In addition, POAF can be triggered also by pericardial effusion. The pericardial effusion stimulates the myocardium and promotes the local inflammatory response, which can lead to POAF. Another recent study found that the posterior pericardial drain can reduce late postoperative pericardial effusion and POAF, compared with the anterior drain, during the first 30 postoperative days (36). To avoid the occurrence of pericardial effusion, we also adopted the the posterior pericardial drain and a mediastinal drainage was placed in the front mediastinum. In addition, we did not suture the pericardium at all, and the pericardium cavity was completely connected to the anterior mediastinum. We only recorded POAF that occurred within postoperative 5 days, no obvious signs of pericardial effusion were found during the period.

Several limitations should be acknowledged in our study. Firstly, the clinical sample size is relatively small, potentially limiting the statistical power of our findings. Subsequent studies with larger sample sizes are warranted to validate our results. Secondly, the propensity score matching process inevitably led to some loss of original data. Lastly, the retrospective study design prevents us from establishing a definitive causal relationship between POAF and adverse outcomes. Consequently, long-term follow-up studies are essential to elucidate whether POAF serves as an indicator or a primary contributor to postoperative adverse outcomes. Although there are some shortcomings in our study, early postoperative hemodynamic stability has an important impact on the recovery of surgical patients supported by extracorporeal circulation, so our findings have certain significance for clinical work.

## 5 Conclusion

In summary, our results indicate that advanced age and LAD are independent preoperative risk factors for new-onset POAF after PEA. New-onset POAF after PEA is closely associated with adverse outcomes. Subsequent large-scale studies are necessary to fully explore the role of POAF in the prognosis of PEA.

## Data Availability

The original contributions presented in the study are included in the article/Supplementary Material, further inquiries can be directed to the corresponding author.
